# Acceptability of malaria rapid diagnostic tests administered by village health workers in Pangani District, North eastern Tanzania

**DOI:** 10.1186/s12936-016-1495-z

**Published:** 2016-08-27

**Authors:** Adiel K. Mushi, Julius J. Massaga, Celine I. Mandara, Godfrey M. Mubyazi, Filbert Francis, Mathias Kamugisha, Jenesta Urassa, Martha Lemnge, Fidelis Mgohamwende, Sigbert Mkude, Joanna Armstrong Schellenberg

**Affiliations:** 1 Centre for Enhancement of Effective Malaria Interventions, 2448, Barack Obama Drive, P.O. Box 9653, Dar es Salaam, Tanzania; 2 National Institute for Medical Research, HQ, 3 Barack Obama Drive, 11101 Dar es Salaam, Tanzania; 3 National Institute for Medical Research, Tanga Centre, P.O. Box 5004, Tanga, Tanzania; 4 National malaria Control Programme, Ministry of Health and Social Welfare, 6 Samora Machel Avenue, 11478 Dar es Salaam, Tanzania; 5 Faculty of Infectious and Tropical Disease, London School of Hygiene & Tropical Medicine, Keppel Street, London, UK

**Keywords:** Malaria rapid diagnostic tests, Village health workers, Acceptability, Rural Tanzania

## Abstract

**Background:**

Malaria continues to top the list of the ten most threatening diseases to child survival in Tanzania. The country has a functional policy for appropriate case management of malaria with rapid diagnostic tests (RDTs) from hospital level all the way to dispensaries, which are the first points of healthcare services in the national referral system. However, access to these health services in Tanzania is limited, especially in rural areas. Formalization of trained village health workers (VHWs) can strengthen and extend the scope of public health services, including diagnosis and management of uncomplicated malaria in resource-constrained settings. Despite long experience with VHWs in various health interventions, Tanzania has not yet formalized its involvement in malaria case management. This study presents evidence on acceptability of RDTs used by VHWs in rural northeastern Tanzania.

**Methods:**

A cross-sectional study using quantitative and qualitative approaches was conducted between March and May 2012 in Pangani district, northeastern Tanzania, on community perceptions, practices and acceptance of RDTs used by VHWs.

**Results:**

Among 346 caregivers of children under 5 years old, no evidence was found of differences in awareness of HIV rapid diagnostic tests and RDTs (54 vs. 46 %, p = 0.134). Of all respondents, 92 % expressed trust in RDT results, 96 % reported readiness to accept RDTs by VHWs, while 92 % expressed willingness to contribute towards the cost of RDTs used by VHWs. Qualitative results matched positive perceptions, attitudes and acceptance of mothers towards the use of RDTs by VHWs reported in the household surveys. Appropriate training, reliable supplies, affordability and close supervision emerged as important recommendations for implementation of RDTs by VHWs.

**Conclusion:**

RDTs implemented by VHWs are acceptable to rural communities in northeastern Tanzania. While families are willing to contribute towards costs of sustaining these services, policy decisions for scaling-up will need to consider the available and innovative lessons for successful universally accessible and acceptable services in keeping with national health policy and sustainable development goals.

## Background

The world has achieved tremendous progress with a 47 % decline in malaria mortality rates and an estimated 4.3 million averted deaths between 2001 and 2013 [1]. Despite this progress, the malaria burden remains heaviest in the African continent where an estimated 90 % of global malaria deaths occur. Such deaths could have been saved if the tools currently available had reached those who needed them [1, 2]. More efforts are urgent to improve access to prompt malaria case diagnosis and management especially in rural African villages where the highest malaria prevalence rate occurs in the midst of inadequate access to health facilities and required human resources for health [3]. Several resource-constrained countries report a positive effect on access to services for malaria and other childhood illnesses after implementing integrated community case management of malaria (iCCM). The iCCM strategy recommended by WHO and UNICEF since June 2012 has successfully used trained community-based service providers to complement health systems in improving access to essential treatment for children, including facilitating prompt management of malaria within 24 h of onset of symptoms [4–11].

Malaria rapid diagnostic tests (RDTs) have the potential to reduce unnecessary and inappropriate treatment of malaria by targeting anti-malarials to only those with confirmed malaria [12–18]. Nevertheless, delivery of known health interventions through the existing health infrastructure remains inadequate particularly in rural areas [1, 19]. Formalization of iCCM with trained village health workers (VHWs) can strengthen and extend the scope of public health services, including diagnosis and management of uncomplicated malaria in areas with insufficient health infrastructure and qualified health workers [1, 18–20]. VHWs, popularly known as community health workers or community owned persons, are a cadre that relies on community members who are trained in delivering malaria case management and other health services in many endemic countries. VHWs deserve attention in the era of sustainable development goals, which include achieving universal access to quality and essential healthcare services [51]. They substantially contribute to improved access to increased demand for basic essential health services, including managing preventive and therapeutic care for malaria and other basic services in compliance with Millennium Development Goal targets [21–23].

VHWs have greatly assisted in overcoming access barriers and increase early treatment-seeking behaviour for malaria symptoms, prompt diagnosis and appropriate prescription of anti-malarials through RDTs, besides facilitating adherence to treatment and hence contributing to reduction in malaria-related mortality in the context of iCCM. Having established convincing evidence in management of malaria and other childhood illnesses, some malaria-affected countries, such as Zambia, Democratic Republic of Congo, Sudan, and Uganda, have reached advanced stages in considering formalization of VHWs [24–29]. Besides treatment, trained VHWs facilitate early referral of malaria parasite-negative cases to health facilities for management by qualified health personnel [19, 20]. Accordingly, countries, such as Ethiopia and Rwanda, have integrated iCCM in their health systems, leading to improved access to essential health supplies, including artemisinin-based combination therapy (ACT), RDTs and antibiotics for pneumonia in line with national guidelines [24–29]. These countries are in the same region and similarly prone to malaria as is Tanzania.

Tanzania is among 16 countries that account for 80 % of malaria deaths globally, as well as being among the 18 countries that account for 80 % of global malaria cases [1]. This situation has prevailed despite long-term initiatives inspired by recognizable political will to fight malaria in 2006 and 2013 in Abuja, Nigeria. Tanzania and other African states resolved to control, eliminate and subsequently eradicate malaria on the continent. The country is among the founders of the African Leaders’ Malaria Alliance (ALMA), a platform that supports accountability mechanisms against malaria in malaria-prone African countries. However, Tanzania has not yet implemented iCCM which is recommended by WHO and listed as one of ALMA’s performance indicators for measuring progress and accountability [30, 31].

The national malaria treatment guidelines in Tanzania recommend confirmatory diagnosis prior to treatment. Tanzania initiated a phased implementation of RDTs in 2009, starting at hospital level, through to health centres and to the dispensary level. Although nearly half (44 %) of the Tanzanian population lives between 2 and 5.9 km from the nearest health centre or dispensary, the prevailing geographical inequalities and resources shortage challenge access to health services in the country in general [32, 33].

The existing ratio of 1.3 health workers per 1000 persons means the country has a shortage of qualified personnel in all cadres, particularly in remote rural areas [34]. Consequently, sub-optimal access to prompt treatment of malaria is likely to persist if service delivery remains through existing health facilities alone [35, 36].

According to lessons from other countries as presented above, formalization of trained VHWs has potential to expand access to malaria diagnosis using RDTs and treatment at community level, in line with policies currently recommended by WHO.

VHWs in Tanzania are usually local people, selected by their own communities and widely recognized as providers of simple, curative and preventive care following swift training/crash courses and subsequent refresher programmes [37]. Tanzania has long experience of community-based health workers who are popularly termed in Swahili, the county’s national language, as *Wahudumu wa afya vijijini*, which translates as village health workers (VHWs). The popularity of VHWs in Tanzania stems from a Tanzanian Government initiative, in collaboration with UNICEF, which introduced the Child Survival, Protection and Development (CSPD) programme during the early 1990s to help conduct village censuses, check child nutritional status and provide support, including nutritional counselling. Since then, VHWs have been involved in broader health service provision than initially envisaged. For example, in southern Tanzania, VHWs have been found working at a government dispensary either together with or sometimes on behalf of trained medical assistants and nurses, in drug prescription and dispensing, besides child immunization and deworming campaigns, child growth monitoring, and community-based family planning services. Some VHWs participating in the present study reported being trained and equipped to administer deworming medicines and provide wound management services in their villages. The unresolved issues around recruitment, training, motivation, incentives, management, and acceptability have previously challenged the sustainability of VHWs in Tanzania [37–40]. It is encouraging to note that VHWs received some consideration in the previous and current national malaria control programme strategic plans [41, 42]. While implementation has not yet started, evidence about the role of VHWs in iCCM in Tanzania is on the rise. Studies conducted in northern and eastern Tanzania have demonstrated the capacity of VHWs to not only apply knowledge received in presumptive treatment of malaria [43], but also to use RDTs to inform decisions to treat positive cases and facilitate referral of those with negative tests to a higher level of care provision. VHWs have proven ability to reduce unnecessary treatment with anti-malarials in parts of Tanzania [44, 45]. Acceptability of a service delivery strategy within targeted communities is a key requirement for scaling up sustainable programmes, including RDTs delivered by VHWs [46, 47]. Here is evidence from rural northeastern Tanzania of community acceptability of RDTs delivered by VHWs.

## Methods

### Study design

A mixed methods, cross-sectional study was conducted between March and May 2012 on community acceptability of RDTs used by VHWs in eight villages within Pangani district, northeastern Tanzania [43].

### Study area and population

Pangani district is located between 5°15.5–6° S and 38°35–39°00 E on the southern coast of Tanga region along the Indian Ocean coastline. Covering an area of 1803.8 km^2^, this rural district had a total population of 54,025 inhabitants in 2012 [34]. The district is characterized by a long rainy season from March to June, and short rains between November and December. There is perennial transmission of malaria, a leading cause of morbidity and mortality among children aged 2–9 years [43]. The main source of income includes subsistence farming, fishing, small-scale livestock rearing, and small trade. A few residents worked on sisal plantations [24].

At the time of conducting this study, Pangani district had a total of 22 dispensaries (19 public and three private), one health centre, one district hospital (public/government), and 15 accredited drug-dispensing outlets (ADDO). In Mwera division where this study took place, there were five health facilities including one health centre, four dispensaries and five ADDOs. Within the same division, there were 15 community-owned resource persons (CORPs) and 48 mother coordinators (MCs). The latter had been introduced in 2007 and equipped with supplies and skills to provide presumptive treatment of malaria to young children within a framework of research on home management of malaria (HMM) [43]. With a grant from a malaria capacity development consortium, a new component was nested within the same framework of the HMM project in Pangani district to study the feasibility of implementing RDTs through existing CORPs and MCs, jointly referred in this paper as VHWs [48]. Under this new component VHWs initially attended a 3-day training course at Pangani district headquarters, followed by attachment to their nearest dispensary or health centre for a period of 1 month. This training equipped them with practical skills to diagnose and treat positive malaria cases confirmed by RDTs, and/or refer negative and sick children to nearby health facilities, as well as record keeping and handling of RDTs before, during and after attending clients. All VHWs attended this training before they received supplies of RDTs and ACT to start services in their villages under close supervision by project team members in collaboration with a field coordinator and those in charge of nearby health facilities.

### Data collection

The quantitative data were collected from eight of the 14 host HMM project villages of Mwera division. These eight villages were purposively selected; half of them had no health facilities to draw a convenient sample of 346 households with children aged under 5 years. In those households, mothers or caregivers of eligible children were interviewed using a structured questionnaire containing questions on knowledge, attitudes, practices, and future prospects of RDTs used by VHWs. Qualitative data were collected through focus group discussions (FGDs) held in four of the eight study villages. Two villages where FGDs took place had a health facility, the remainder did not. In each village, two FGDs were held, one with mothers or caregivers, and the other with VHWs to investigate awareness, willingness to pay, perceptions, practices, acceptability, and future prospects regarding RDTs used by VHWs.

### Demonstration of RDTs

Prior to data collection, it was possible for respondents to see RDTs during FGDs, household surveys and concurrent community malaria surveys. Some respondents had also seen RDTs at nearby local health facilities or in use by VHWs in the study communities.

### Language used

Swahili, the national language of Tanzania, is usually spoken in the area and was used throughout data collection.

### Data management and analysis

Quantitative data were double-entered in Epi-info version 6.1 (CDC) software to check logical completeness and consistencies before analysis was performed according to a pre-defined analytical plan, using Stata version12 (Stata Corp LP, College Station, Texas, USA). Chi square tests were used to assess the association between categorical variables. p value was considered significant at <0.05.

The FGDs were recorded using MP3 voice-recorders and transcribed verbatim alongside daily debriefing notes. All transcripts were manually scrutinised to code-related text under corresponding themes that generated the qualitative excerpts presented in this paper.

### Ethical considerations

This study received ethical approval certificate number NIMR/HQ/R.8a/Vol.IX/1670 from the Medical Research Coordinating Committee of the National Institute for Medical Research (NIMR) in Tanzania. The district and village authorities were sensitized about the study before granting permission to work in their areas. Written informed consent was obtained from all study participants.

## Results

Information on the type of data collected, data collection techniques, areas covered, and total data collected per technique is summarized in Table 1. In total, 346 mothers/caregivers of under-5 children with median age of 29 years (IQR 22–35) participated in the household survey. A total of four separate FGDs, each with eight participants was held with 32 FGDs in four villages. Equal numbers of mothers of under-5 children and VHWs from villages with and without VHWs participated in these sessions. The majority of household respondents had completed primary education (Table 2).

**Table 1:** Data collection summary

Type of data collected	Data collection techniques	Participants	Number of villages covered	Total data collected
Quantitative	Household questionnaire	Mothers of children aged less than 5 years	8 out of 14 HMM study villages (4 with, and 8 without health facilities)	346 household questionnaires administered
Qualitative	Focus group discussions	Separate sessions for mothers of children aged less than five years and village health workers	4 out of 8 villages where mRDTs component took place (2 with, and 2 without health facilities)	8 FGD sessions (two separate FGDs in each village, each with mothers/VHWs)

The host HMM project that relied on presumptive treatment of malaria covered 14 villages. The current study with mRDTs component took place in 8 out of the 14 HMM study villages

**Table 2:** Demographic characteristics of mothers/caretakers

Variables (n = 346)	n	%
Sex		
Male	23	6.7
Female	323	93.3
Median age (IQR) in years	29	22–35
Age distribution in years		
14–24 years	114	33
25–34 years	145	42
35+ years	87	25
Education		
Primary	277	80
Secondary	21	6
Informal	3	1
None	45	13
Marital status (N = 345)		
Single	45	13
Married	272	79
Separated	13	4
Widow	4	1
Divorced	11	3
Caregivers		
Village with health facility	161	46
Village without health facility	183	53

### Awareness of caregivers on different types of RDTs

**Fig. 1:**
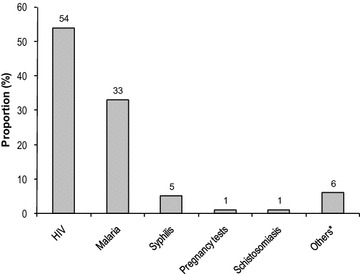
Awarenes of different types of rapid diagnosis test

The respondents were firstly asked several questions on RDTs in order to situate RDTs in their existing understanding and experience. A total of 346 caregivers responded on awareness of RDTs used for HIV screening, RDTs and other diseases. No evidence was found of a difference in awareness of RDTs used for HIV and those used for malaria (54 vs. 46 %, p = 0.134), with very few reports of RDTs used for other health purposes (Fig. 1). In FGDs, mothers/caregivers more often mentioned RDTs for HIV used at health facilities than RDTs, based on the knowledge they gained particularly from the district hospital. Some participants confidently reported personal experience of being screened for HIV using similar tools and approach as they had seen used in a community malaria survey.

As an indication of positive attitudes towards RDT use in HIV testing, some FGD participants expressed a wish for similar tests for malaria in their community setting:

*We encourage the government to introduce RDTs in health facilities and to our VHWs as they did for HIV RDTs because this will help us to know if our children have malaria, to what extent. If not we can be advised on where else to seek care instead of receiving malaria treatment presumptively.* (FGD, Mothers, village 2)

*There is no one who does not know about RDT for HIV AIDS these days. If you go to Pangani district hospital for mother and child health services don’t get surprised to be invited for a blood check*-*up using RDT together with your sexual partner. It is a nice thing and if introduced for malaria I will go for that personally and my child because malaria parasites will be checked and results obtained within short time.* (Mother, FGD, village 2)

### Caregivers’ readiness to accept RDTs if administered by VHWs

Caregivers were sequentially asked to express readiness to accept the RDTs administered by VHWs for themselves, or for their children in case of malaria symptoms. The majority (96 %) of 346 interviewed caregivers stated readiness to accept RDTs in both scenarios. As shown (Table 3), readiness to accept RDTs was significantly higher among caregivers with formal education 97 % (95 % CI 95–98 %) compared to those without formal education 90 % (95 % CI 81–99 %, p = 0.02). There was no evidence of an association between readiness to accept RDTs and either (i) the distance from home to the nearest health care facility; or, (ii) gender, age or marital status. Similarly, the majority (96 %) of caregivers indicated readiness to accept the RDTs for their own child if administered by VHWs.

**Table 3:** Willingness to accept mRDTs if administered by VHWs

Variables	Willingness to accept mRDTs		p value
	Yes	No	
Availability of health facility, n (%)			
Community with health facility	156 (97)	5 (3)	0.407
Community without health facility	176 (95)	9 (5)	
Age groups in years, n (%)			
14–24 years	110 (97)	4 (4)	0.454
25–34 years	137 (95)	8 (6)	
35+ years	85 (98)	2 (2)	
Sex, n (%)			
Male	23 (100)	0 (0)	0.308
Female	309 (6)	14 (4)	
Marital status, n (%)			
Single	43 (96)	2 (4)	
Married	262 (96)	10 (4)	0.836
Separated	12 (92)	1 (8)	
Widow	4 (100)	0 (0)	
Divorced	10 (91)	1 (9)	
Education level, n (%)			
Formal (primary school or above)	289 (97)	9 (3)	0.02
Informal (never been to school)	43 (90)	5 (10)	

### Perceived benefits of RDTs administered by VHWs

Respondents’ perceived benefits of RDTs used by VHWs included confirmation of presence or absence of malaria parasites before prescription (51 %), brings diagnostic services closer to community (36 %) and receiving results more quickly (9 %).

The majority of respondents (91 %) among the 346 caregivers in the household survey felt that RDTs would increase utilization of services provided by VHWs. However, 7 % of respondents reserved their opinion while (2 %) avoided RDTs used by VHWs due to perceptions that VHWs might not be competent enough to perform such services.

Mothers in this study thought that introduction of RDTs would add value to the services rendered by VHWs and would complement those provided at health facilities, which were initially perceived as inadequate due to a lack of diagnostic services. Mothers supported implementation of RDTs through VHWs with expectations for improved services.

*We already know them [VHWs]. Mothers have been presenting their children to receive Mseto [i.e. ACT], only based on their thermometer and weighing scale which provides weight to guide on the amount of required drug. So, adding RDTs will increase people’s respect to their services because anyone would like to see her child getting the right treatment for a confirmed problem.* (Mother, FGD, village 1)

*What we dislike is how our doctors at dispensaries and health centres endanger lives by giving anti*-*malarials to children before screening them to know problem. You just see them opening a box with Mseto [ACT] they give you a dose with instructions to follow when administering to a child. If symptoms persist, and you take the child there again they will give quinine, and in case of recurring symptoms they instruct to go further to the Pangani district hospital. This makes us doubt about quality of the malaria treatment services given to our children. None of us would say no to RDTs if introduced to our VHWs because that will give us and our children what health facilities do not have*. (Mother, supported by all the members in the group, FGD, village 2)

*Some of us initially doubted these VHWs when we heard that they would be testing blood to know if a child deserves anti*-*malarials or not. It is not a joke when it comes to being able to diagnose malaria and give medicines. But now, I wish they will continue. I am saying so because what they are doing is not common for someone without advanced secondary school or nursing qualification. I will support if we can get even more VHWs in our sub villages as long as they will handles like the one we know. We have seen them receiving practical training before they come to community. And our doctors and nurses have assured us that the VHWs had qualified training. Various people who went to dispensary also spread news that the VHWs were practicing like experienced health workers. The same doctors also refer us to VHWs with our children sometimes for malaria diagnosis or medicines during stock outs at the dispensaries.* (Mother, supported by all the members in the group, FGD, village 2)

### Decision-making on the use of RDTs from VHWs

Over half (58 %) of all respondents in the household survey thought that mothers could lead decisions in seeking services, including those involving the use of RDTs from VHWs, although at times fathers could also do so while 30 % of them said that the head of a household or other guardian could make a decision to take a sick child to the VHW depending on the perceived urgency. Very few (12 %) thought that others in the family could make such a decision in the absence of the parents.

### Prerequisites for accepting RDTs administered by VHWs

The caregivers were asked to comment on “what should be done to ensure successful use of RDTs if administered by VHWs?” The most common response (38 %) was the need for training of the VHWs on the administration of this technology, followed by those suggesting the need for ensuring reliable availability of anti-malarials and RDTs (29 %). Others recommended supportive supervision (9 %) of VHWs coupled with community sensitization (8 %). The respondents suggested communication channels about RDTs administered by VHWs should include the use of a village crier, in Swahili *Mpiga Mbiu,* (48 %), a village assembly (28 %), radio (7 %), posters, leaflets and religious leaders (20 %).

### Caregivers’ perceived willingness to pay for RDTs administered by VHWs

The overwhelming majority (92 %) of household respondents (n = 346; 95 % CI 89–95 %) said they were willing to pay for RDTs administered by VHWs. In a question about what they would be willing to contribute towards the costs of RDTs used by VHWs, the median cost mentioned was Tshs 6000 (approx US$3), ranging from Tshs 500 (US$0.4). FGD participants also gave their views in favour of contributing to costs of RDTs used by VHWs:

*The test [RDT] is more important than ACT which we have been receiving from VHWs and hospitals without knowing the exact problems in our children. There are plenty of such medicines even at local shops at very low price (Tshs 500). What is most important is the diagnostic test and not relying on the thermometer only, and therefore we better have it even if it will be done at a small charge*. (Mother, FGD, village 2)

## Discussion

Can rural communities in Tanzania accept RDTs if implemented by VHWs? This study took place when Tanzania had a policy for using RDTs, not only for malaria screening at hospitals but for other diseases, including HIV and syphilis [49]. Hence, in order to situate discussions in existing realities, interviewing was extended to explore local knowledge and perceptions of rapid diagnostic tests used in HIV and syphilis besides malaria. Taking into consideration the broader notion of fears and stigma attached to HIV/AIDS testing in the past [50], and similarity of RDTs and RDTs used for HIV screening, it was considered necessary to understand community perceptions on such tests. Accounts from the majority of mothers and VHWs who shared positive personal experience of RDTs in HIV screening, did not only allow continuation of in-depth discourses that revealed high readiness of community members to accept RDTs, but also paved the way to adapting questions and discussions about RDTs used by VHWs into local understanding and practices about similar health interventions. This initial entry point was considered necessary. Knowledge about local perceptions of local health services is a key to scaling up malaria interventions, including RDTs [51]. Moreover, implementation of new interventions or clinical guidelines may not be successful, based on evidence, if it does not consider the crucial role of local knowledge and context [52, 53].

The community in this study expressed acceptance of RDTs after hearing that VHWs attended training at district headquarters and later at local health facilities, or in direct encounter during practical training at a nearby dispensary or health centre. Such acceptance might not have been possible in this study where villagers did not expect someone without secondary school education or nursing background to be able learn and perform sensitive tasks such as using RDTs and giving medication to children. Hence, what appears as believing and accepting after seeing, community attitudes changed towards VHWs, inspired not only by knowledge about VHWs having attended training at district headquarters but also by actual experience of their services during subsequent practical training at local health facilities before they started applying RDTs in their villages. The same VHWs were already accepted in provision of presumptive malaria management services in the same areas, and equipping them with skills and supplies transparently contributed to acceptability of RDTs.

The findings have also shown approval of VHWs from health workers at local dispensaries and health centres. This together with situations in which VHWs referred sick children to health facilities after negative results from RDTs and vice versa, in the case of RDTs and ACT stock-outs at health facilities, portrayed good relationship between formal health workers and VHWs. Health facility staff are trusted as reliable source of health information in Tanzania [54]. Their approval and support, and their good relationship with VHWs are potential elements that boost community acceptance of RDTs used by VHWs has also been shown to inspire acceptance of tuberculosis therapy in Tanzania [55].

Availability of medication usually results in increased client satisfaction with health services in national community health worker programmes [38, 56]. In this study, community members appreciated reliable availability of RDTs and anti-malarials from VHWs. Moreover, the community reported convenient access to VHWs as an aspect that promoted mutual respect and understanding of service provision and utilization.

A few mothers/caregivers in this study indicated that they might not take their children to VHWs even if they were equipped with RDTs, because of perceived limited ability of fellow villagers to master professional tasks. This raises questions such as, what might happen if a child felt sick in an area far from a health facility but where there is a VHW with RDTs? Would those parents necessarily avoid RDTs used by VHWs and opt for the distant health facility if they suspected malaria? The answer to this health seeking puzzle might link to existing knowledge of similar services. Refusals among parents/caregivers of children aged under 5 in consenting for earlier diagnosis has been documented in health facility-based studies conducted in Tanzania and other countries. This could happen due to perceptions that malaria symptoms are easily recognized and that anti-malarials are known to them and readily accessible for self-treatment. Perceived ability to recognize malaria symptoms among parents/caregivers might lead them to put pressure on VHWs as they have earlier done with health facility staff on prescription of anti-malarials. Parents may as well justify avoiding RDTs for their children if the costs are higher than alternative sources for managing “fake malaria” [57–60]. Regardless of whether RDTs are to be used by VHWs or not, there is a need to consider tailoring behaviour change communication on RDTs to convince potential users on where to get them affordably as well as the benefits of not relying on symptoms alone to diagnose malaria [58].

Promisingly, on balance, the perceived benefits outweighed the negative perceptions as the majority of respondents in this study expressed readiness to accept RDTs administered by VHWs for themselves, or for their children, in case of malaria symptoms. Presence of VHWs with RDTs and ACT in villages without health facilities with reduced travel to health facilities is consistent with the call for improving access to fever case-management through RDTs [61]. As in other places, mothers and VHWs in this study commented on the benefits of RDTs used by VHWs in terms of reducing the budget associated with over-use of ACT in children with negative malaria results, as well as controlling attendance to already resource-constrained health facilities, which could be costly to reach due to travel costs [16, 31, 34, 35, 62]. Mothers also linked RDTs used by VHWs with reducing drug costs which could be incurred based on clinical judgment as reported from studies conducted on the routine healthcare system [18, 19]. Tanzanian health workers have shared views in favour of RDTs to avoid over-use of ACT, both for reasons of reducing cost and for fear of anti-malarial resistance [58].

### Lessons for implementation

The Ministry of Health in Tanzania requires evidence on the applicability of RDTs at community level [42]. Lessons from Uganda suggest the necessity of strategies to improve drug supply, community support and feedback provision from the formal health system for better performance by community health workers [63]. As well as training and regular supplies, the findings have highlighted a need for close supportive supervision of VHWs in order for them to provide acceptable services [64], otherwise, community members may lose faith or trust in poorly trained VHWs when it comes to medical competence [65]. In Uganda, the iCCM became acceptable to caregivers after they learned that community-based health workers had received training on how to use RDTs and make appropriate decisions [66]. When communities hear about VHWs using RDTs they are likely to apply a ‘seeing is believing’ approach in making judgement about trusting this strategy in the knowledge that they have attended appropriate training, including practical sessions at local health facilities. Approval from health workers based at local health facilities and good relationship with VHWs also boosted community acceptability of VHWs and RDTs that that they use in the villages. Success of RDTs relies on the efforts to ensure community comprehension and trust in the skills of VHWs alongside initiatives to motivate these practitioners beyond the long-term tradition of regarding them as volunteers [67].

Children aged under 5 years are exempted from user fees for health services in the Tanzanian national health policy [68]. Parents/caregivers were not required to meet the costs of RDTs and ACT for children targeted in this study [47, 58]. Nevertheless, a previous multi-country study that included Tanzania reported parents’ willingness to pay not more than Tshs 500 (approx US$0.4) on ACT [58]. This price is consistent with the lowest price that this study recorded on RDTs. The highest reported willingness to pay on RDTs in this study, Tshs 6000 (approx US$3), was twice that of the typical RDT sold in a pharmacy in Dar es Salaam, the commercial capital of Tanzania, of Tshs 3000 (approx US$1.5). To make RDTs accessible to around 90 % of Tanzanian consumers, the recommended price was Tshs 250 (approx US$0.20–0.25) [58]. Willingness to pay may not necessarily imply actual ability to pay [26], and given the existence of socio-cultural barriers, may discourage utilization, and therefore the reported willingness to pay in this study should be viewed cautiously [57–60]. Government and donor willingness to commit funds is an option to make RDTs affordable if the intervention is to be used to significantly reduce the burden of malaria, especially for the poorest consumers and vulnerable members of the society, most of whom live in rural villages [58].

Policy decisions on sustainability of RDTs used by VHWs are expected to be aligned with Sustainable Development Goal 3, which includes a target to achieve universal health coverage through equitable access to affordable, essential medicine and vaccines. This will require integration of individual and population-level health promotion and preventative efforts with curative services [8, 69]. The lessons from possible barriers associated with willingness to pay and other socio-cultural barriers may inspire further in-depth, socio-economic, effectiveness studies on how to ensure sustainable financing, availability, increased coverage, and utilization of RDTs, not only through VHWs, but also by government and private healthcare providers [12, 47]. Recognition of services delivered by VHWs and integration of these services into the national health delivery policy could pave the way to supply chain and management in line with available guidelines. In this study, they complied with provision of free services to the target group of children aged under 5 years according to national policy. Nevertheless, Tanzania could learn from past local experience and best practices from countries where VHWs are involved in iCCM. Innovative sustainability strategies will be required to better address critical issues such as alignment of VHW programmes with existing public health system, effective management strategies, motivation, incentives, support from health workers based at nearby health facilities, uninterrupted availability of supplies, affordability to targeted users, monitoring and evaluation, and advocacy to promote the use of RDTs [4–6, 9, 10, 67].

### Strengths and limitations

The study included areas located far from health facilities in a district where people are generally highly aware of malaria as a public health problem. Health facilities in the study area had limited supplies and service providers, and some were open for 12 h or less each day. These factors might have led to the high acceptability of RDTs reported to a level that might not be duplicated in areas with different coverage of healthcare services. The present study was nested within a framework of a long-term research on HMM, which is not typical of Tanzania. As for the representativeness of the national situation, a study done in only one district is likely to provide limited data and evidence for generalization for the whole of Tanzania. There is a room to argue on limited generalizability of the study findings from a quantitative point of view. Qualitatively, this approach may not be sufficient to facilitate in-depth understanding, which might need more time and a different approach, such as participant observation [70, 71].

However, the study attempted to minimize limitations by involving a competent multidisciplinary team at all stages. It exposed community appreciation of the role of VHWs in malaria case management both presumptively and by using RDTs in under-served populations according how they received training and supplies.

## Conclusions

RDTs implemented by VHWs are acceptable to rural communities in north eastern Tanzania. While families are willing to contribute towards the costs of sustaining these services, policy decisions for scaling-up will need to consider the available and innovative lessons for successful universally accessible and acceptable services in keeping with national health policy and sustainable development goals.

## Abbreviations

ACT: artemisinin-based combination therapy
ADDO: accredited drug dispensing outlets
ALMA: African Leaders’ Malaria Alliance
HMM: home management of malaria
CORPs: community-owned resource persons
CSPD: Child Survival, Protection and Development programme
FGD: focus group discussion
iCCM: integrated community case management of malaria
MCDC: Malaria Capacity Development Consortium
MCs: mother coordinators
RDT: rapid diagnostic test
UNICEF: United Nations Children’s Emergency Fund
VHWs: village health workers
WHO: World Health Organization
